# Plants expressing murine pro-apoptotic protein Bid do not have enhanced PCD

**DOI:** 10.1186/s13104-020-05285-x

**Published:** 2020-09-21

**Authors:** Anna Manara, Zahra Imanifard, Linda Fracasso, Diana Bellin, Massimo Crimi

**Affiliations:** Department of Biotechnology, University of Verona, Strada Le Grazie 15, 37134 Verona, Italy

**Keywords:** Programmed cell death, Apoptosis, BH3-only protein, Bid, Oxidative stress, Nitric oxide, Plant pathogen

## Abstract

**Objectives:**

The purpose of this study was to explore whether plant programmed cell death (PCD) cascade can sense the presence of the animal-only BH3 protein Bid, a BCL-2 family protein known to play a regulatory role in the signaling cascade of animal apoptosis.

**Results:**

We have expressed the mouse pro-apoptotic protein Bid in *Arabidopsis thaliana* and in *Nicotiana tabacum*. We did not obtain any transformed plant constitutively expressing the truncated protein (tBid—i.e. the caspase-activated form) whereas ectopic expression of the full-length protein (flBid) does not interfere with growth and development of the transformed plants. To verify whether the presence of this animal pro-apoptotic protein modified stress responses and PCD execution, both *N. tabacum* and *A. thaliana* plants constitutively expressing flBid have been studied under different stress conditions triggering cell death activation. The results show that the presence of flBid in transgenic plants did not significantly change the responses to abiotic stress (H_2_O_2_ or NO) and biotic stress treatments. Moreover, the finding that no Bid active form was present in treated tobacco plants suggests an absence of a proper activation of Bid.

## Introduction

Programmed cell death (PCD) is present both in animal and plant systems, where different modes of action have been recognized and studied. Among these, apoptosis and apoptotic-like PCD differentiate from other forms of cell death and share common morphological characteristics.

In animals, apoptotic cell death involves the Bcl-2 protein family as the initiator of the signaling pathway and a cascade of caspases for the execution of the death program [1]. So far, no Bcl-2 proteins have been identified in plants. Nevertheless, there is evidence that Bcl-2-like proteins exist. In 2001, a gene encoding for a homolog of the human gene BI-1 was isolated from Arabidopsis (AtBI) [2]. The existence of a functional Bax Inhibitor in plant indicated the possibility of a Bax-like mechanism in plants or the presence of a regulatory system similar to that of animals (i.e. pro- and anti-apoptotic mediators). Moreover, when the anti-apoptotic gene Bcl-XL was expressed in tobacco, death triggered by inducers of cellular ROS like UV, paraquat or TMV, was suppressed [3]. In addition, the expression of anti-apoptotic genes (Bcl-Xl and Ced-9) in tomato plants improved tolerance to viral infection and low temperature exposure [4]. These results indicated that a conserved apoptotic pathway might be present in plants with divergent proteins.

In order to study the presence of a common signaling pathway in the sequence of cellular processes occurring during PCD, we have expressed mouse Bid, a pro-apoptotic BH3-only protein, in plant systems. This protein is located at the crossroad of the extrinsic and intrinsic signaling pathways of apoptosis, driving death signal to mitochondria through interactions with pro- and anti-apoptotic proteins [5]. Bid exists as a precursor (full-length protein, flBid) and it becomes activated (truncated Bid, tBid) upon cleavage by Caspase 8 [6]. Other mechanisms, triggered by different stimuli, influence Bid activity such as the reversible binding with lysolipids or cardiolipin.

In the present study, we report the generation of transgenic *Arabidopsis thaliana* and *Nicotiana tabacum* plants expressing the mouse Bid protein and the response of transgenic plants to abiotic and biotic stresses.

## Main text

### Materials and methods

#### Plant material and growth conditions

*Arabidopsis thaliana* wild type (Col-0), *Nicotiana tabacum* cv. Petit Havana SR1 and transgenic seeds were sown in Petri dishes on water-soaked Whatman filter paper and incubated for 3 days at 4°. Plants were grown under controlled greenhouse conditions (100-120 μmol m^−2^s^−1^, 16/8 light/dark).

For in vitro cultivation, plants were grown into Petri dishes containing the MS [7] medium and placed in a growth chamber (16 h light/8 h dark, 22 °C/18 °C 80-120 μmol m^−2^ s^−1^).

#### Generation of flBid and tBid over-expressing Arabidopsis thaliana and Nicotiana tabacum lines

To create a Cauliflower Mosaic Virus (CaMV) 35S plant expression construct, the 588 bp long *flBid* and the 414 *tBid* coding sequences (GeneBank accession number MMU75506, https://www.ncbi.nlm.nih.gov/nuccore/U75506.1) were amplified from mouse cDNA by PCR using sequence specific primers (Additional file 1). The amplified sequences were inserted into the expression vector pH2GW7 by LR reaction (Gateway^®^ LR Clonase™, Invitrogen). The resulting constructs were introduced by electroporation into competent *Agrobacterium tumefaciens* strain GV3101 pMP90RK [8]. *A. thaliana* Col-0 wild type plants were transformed by floral dip [9]. *N. tabacum* cv. Petit Havana SR1 wild type plants were transformed by *A. tumefaciens* cocultivation. Transformed plants were selected on MS [7] medium containing hygromicin. Transgene insertion was verified by PCR and protein over-expression/truncation was confirmed by western blotting with the specific human/mouse Bid polyclonal anti-Bid antibody (R&D Systems). Seeds were harvested and homozygous plants were screened.

#### DNA laddering analysis

Five micrograms of extracted genomic DNA (DNeasy Plant Mini Kit Qiagen) were run for 60 min at 50 V on 1.0% agarose gel.

#### Abiotic stress analysis

##### Oxidative stress experiments

For tobacco, discs of about 1 cm diameter were excised from healthy and fully expanded leaves and floated in a water containing different concentrations of H_2_O_2_ for 24 h. For Arabidopsis leaf discs (6 mm diameter) from 6-week old plants were floated on water for 30 min. H_2_O_2_ was added to 30 mM and conductance measured after 16 and 24 h by using the B-173 compact conductivity meter (HORIBA) as described [10]. For analysis of roots, five-day old seedlings were incubated in 25 mM H_2_O_2_ for 5 min and observed after 24 h to detect apoptotic-like PCD symptoms [11].

##### Nitric oxide fumigation

Four weeks old Arabidopsis plants were fumigated for 8 h under dark conditions with NO diluted in air at the given concentrations by using a fumigation system as described [12] to trigger cell death activation. NO concentration was validated by a chemiluminescence-based NO detector machine CLD70E (Eco physics^®^). Surviving plants were counted 7 days after treatment.

##### Biotic stress analysis

HR electrolyte leakage assay to quantify avirulent pathogen induced cell death was performed as described in [13].

### Results and discussion

#### Expression of tBid in plants is lethal while flBid is not

From the *A. thaliana* stable transformation, 8 transformed plants containing the *flBid* coding sequence and only 2 transformed plants containing the *tBid* coding sequence were obtained. For *N. tabacum*, there were 13 transformed plants with the *flBid* coding sequence but only 3 with the *tBid* sequence. The expression and the presence of the Bid protein were assayed by western blot performed on the total leaf proteins using a polyclonal anti-Bid antibody able to detect both the full-length and the truncated forms (Additional file 2). All the *A. thaliana* and *N. tabacum flBid* transformed plants expressed the gene and accumulated the full-length protein in leaves. In contrast, none of the recovered plants transformed with the truncated form expressed, neither at very low level, the tBid protein. This suggests that the truncated form tBid is able to induce premature cell death in plants preventing the regeneration of transformed plants.

Three stable transgenic *A. thaliana* and three *N. tabacum* plants (35S::*flBid*) expressing the flBid protein at higher levels were selected. Wild-type plants transformed with the empty pH2GW7 expression vector were used as control (WT). Transgenic *N. tabacum* and *A. thaliana* 35S::*flBid* showed normal growth, germination rate and seed production. These observations suggest that the presence of Bid protein in its full-length form is not able to induce cell death in plants and does not interfere with normal growth and development in the model plants utilized here.

#### H_2_O_2_ treatment of wild-type and Bid expressing tobacco plants

To explore if the presence of flBid can interact with the plant cell death signalling pathway either accelerating the process or inducing resistance to stress, both *A. thaliana* and *N. tabacum* transformed plants constitutively expressing *flBid* were analyzed under stress conditions known to be able to induce programmed cell death activation.

*Nicotiana tabacum* leaf discs were treated with hydrogen peroxide (H_2_O_2_) at different concentration (5, 10 and 20 mM) and for 24 h. H_2_O_2_ was chosen because it can act as a secondary messenger both in cell signalling and during the PCD in response to pathogen attack (HR) [14].

**Fig. 1:**
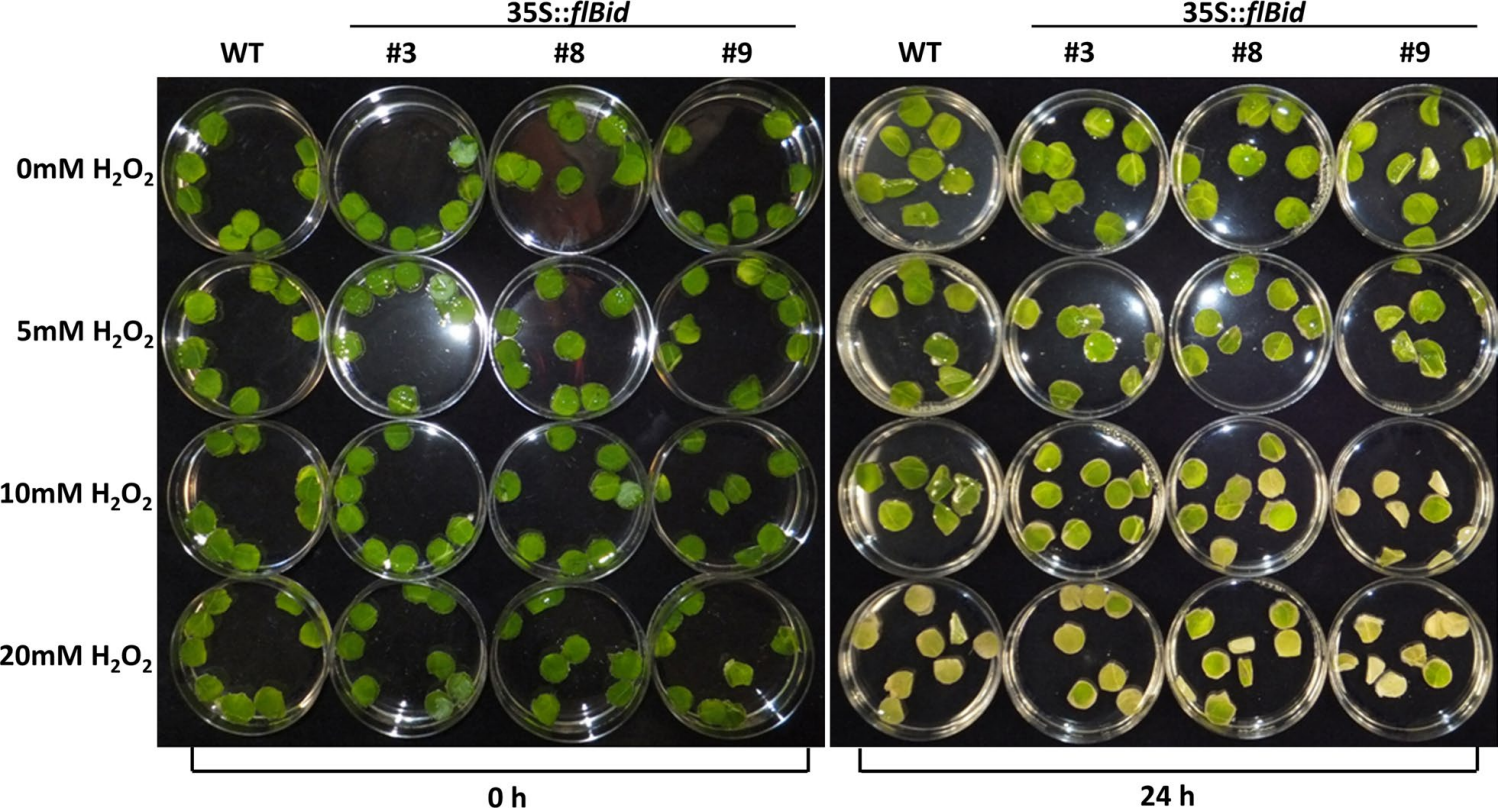
Wild-type (WT) and transgenic (35S::*flBid* lines 3, 8, 9) *N. tabacum* leaf discs in the absence (0 mM) and in the presence of different H_2_O_2_ concentrations (5, 10 and 20 mM) after 0 h (**a**) and 24 h (**b**)

Tobacco leaf discs were observed to evaluate chlorosis and cell death. Leaf discs prepared from 35S::*flBid* plants showed a similar pattern of chlorosis and cell death to untransformed. The only weak difference observed was in the 10 mM treatment where a higher level of chlorosis seems to be present in flBid leaf discs when compared with WT plants (Fig. 1).

The genomic DNA extracted from leaf discs treated with H_2_O_2_ was analyzed to evaluate changes in the time course of DNA fragmentation (DNA ladder, a distinctive trait of apoptosis and programmed cell death) at 0, 1, 3 and 6 h of treatment. No differences were observed between the three lines transformed and the wild type tobacco plants (Additional file 3) and no DNA ladder formation was found.

In addition, we tried to evaluate the formation of tBid after oxidative stress induced by H_2_O_2_ in leaf discs. The analysis did not reveal the appearance of the truncated protein following H_2_O_2_ treatment in transgenic lines (Additional file 4), suggesting that there is no animal-like Caspase cleavage activity to process the Bid protein.

#### Nitric oxide fumigation of wild-type and Bid expressing Arabidopsis plants

A fine balance of reactive oxygen intermediates and NO is involved in the activation of PCD during the hypersensitive response to avirulent pathogens [15, 16]. In the absence of pathogens, this can be mimicked by exogenous NO treatment [17]. To further enquire whether flBid expression in plants could interact with the cell death activation triggered by external PCD inducing stimuli we treated WT and *35S:flBid* Arabidopsis homozygous T3 plants with nitric oxide gas at 160 ppm and 200 ppm in air. As shown in Table 1, no significant difference was found in the response to NO treatment at both NO concentrations between transgenic and wt plants. Therefore, the presence of flBid protein did not interact with the cell death signaling induced by NO. These data indicate that likely no activation of a caspase-like cascade and no cleavage activity similar to that present in animals for processing the Bid protein is present in this system as seen in tobacco, although the absence of a tBid form was not directly enquired.

**Table 1:** Percentage of plants surviving NO treatments

	160 ppm NO		200 ppm NO	
	% surviving plants	*t* test	% surviving plants	t-test
*35S:flBid* replicate1	8.0	n.s.	3.8	n.s.
*35S:flBid* replicate2	12.2		1.4	
*35S:flBid* replicate3	11.0		1.3	
Wild type replicate 1	11.3		3.3	
Wild type replicate 2	6.5		1.3	

Each replicated experiment included at least 240 plants. A statistical t-test was conducted to assess significance of observed differences

#### Avirulent pathogen treatment of wild-type and Bid expressing Arabidopsis plants

**Fig. 2:**
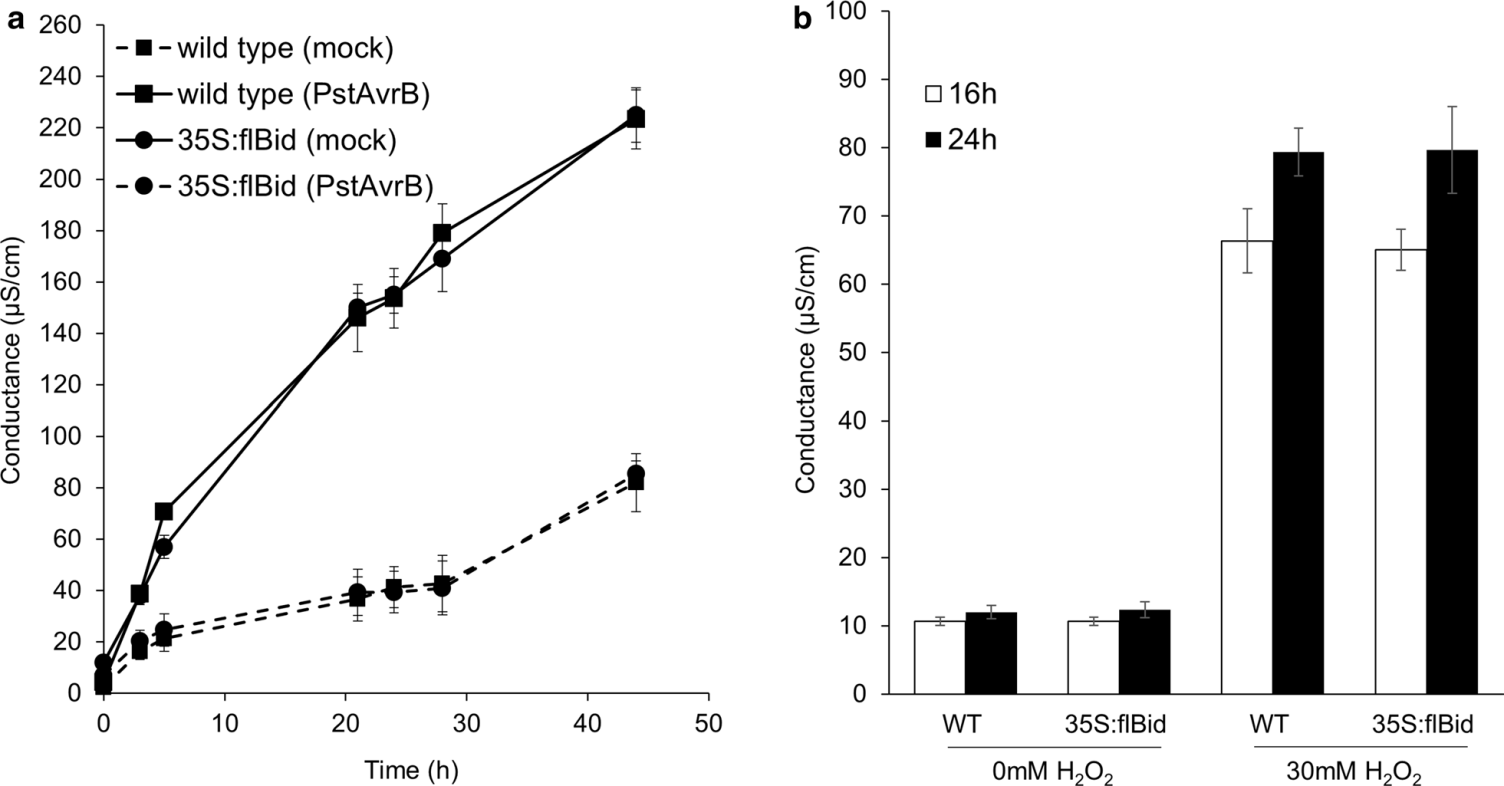
Electrolyte leakage from leaf discs of wild type and *35S::flBid* Arabidopsis plants either **a** infiltrated with *PstAvrB* (10^7^ cfu mL^−1^) or **b** treated with 30 Mm H_2_O_2_. Experiments were repeated three times with similar results

Treatment with the avirulent pathogen *Pseudomonas syringae* pv. *tomato* carrying AvrB (*PstAvrB*) induce the activation of an hypersensitive response in plants which is accompanied by the induction of a programmed cell death at the infection site which aims to restrict pathogen growth and spread [18]. To further enquire whether flBid expression could affect program cell death establishment we treated WT plants and plants expressing flBid protein with *PstAvrB*. Cell death establishment was measured for 48 h by an optimized ion leakage assay [13]. However, no significant difference was found in *35S:flBid* compared to wild-type plants (Fig. 2a) demonstrating that flBid protein does not contribute to the plant PCD occurring during the HR.

#### H_2_O_2_ treatment of wild-type and Bid expressing Arabidopsis plants

Finally, *A. thaliana* plants constitutively expressing *flBid* were also analyzed under H_2_O_2_ treatment conditions known to be able to induce PCD activation, similarly as done for tobacco plants, to determine if there is any flBid cross-talk.

PCD triggered by H_2_O_2_ in leaves was measured following treatment with 30 mM H_2_O_2_ in leaf discs [10]. No differences were found in the *35S:flBid* answer compared to wild type (Fig. 2b).

Moreover, the induction of programmed cell death was further analyzed in roots in *A. thaliana* plants constitutively expressing the flBid protein, after treatment with H_2_O_2_ with 1, 2, 5 and 10 mM H_2_O_2_ for 24 h which can activate apoptosis [11]. The presence of changes in the cells of root hairs was observed under the microscope to detect root hair protoplast contraction and vitality (by FDA loading). It was found that *A. thaliana* flBid lines, after H_2_O_2_ treatment, exhibit a reduced number of alive root hairs compared to wild type demonstrating that flBid leads to higher sensitivity to abiotic stresses in transformed plants, but the effect of the protein in these plants is different from PCD activation, as observed in tobacco leaf discs treated with H_2_O_2_ (Fig. 1). This is likely due to an increase H_2_O_2_ stress sensitivity (i.e. necrosis) since we found apoptosis was not affected (Additional files 5 and 6) thus confirming what we observed in tobacco leaves.

### Conclusions

We have expressed mouse Bid pro-apoptotic BH3 protein in plants to enquire for common signaling pathway during PCD in animal and plants. Our data showed that the active Bid protein (tBid) didn’t allow us to recover any transformed plant, suggesting that the expression of this protein is not compatible with normal plant growth. Furthermore, we also enquired the effect of the ectopic expression of full length Bid protein in plants applying different stress conditions triggering PCD. The results showed that biotic and abiotic stress treatment didn’t lead to any significant change in PCD activation. All together these data suggested that a proper Bid processing activity induced by applied stress is missing in plants.

## Limitations

Expression in two different plants systems of the active truncated Bid protein interestingly did not allow us to recover any transgenic line on the contrary of flBid. However, despite several biotic and abiotic stress treatments applied we couldn’t successfully activate the flBid protein in the plant system to finally demonstrate the presence of a plant target for the active Bid.

## Supplementary information

**Additional file 1.** Forward and reverse PCR primers. Starting and stop codons are underlined, CACC sequence for the pENTR-TOPO subcloning is reported in bold.

**Additional file 2.** Western Blot analysis of flBid and tBid expression in A. thaliana (A) and N. tabacum (B) transformed plants. Numbers represent different transformed lines (1-2-3-4-5-7-8-9-11 for flBid and 2-3 for tBid); rBid: purified recombinant Bid protein; WT: wild type plants extract.

**Additional file 3.** DNA laddering of tobacco leaf discs treated with 0, 5, 10 and 20 mM H_2_O_2._

**Additional file 4.** flBid and tBid Western blot analysis. Total proteins extracted from *wild type* (WT) and 35S::*flBid* (lines 3, 8, 9) *N. tabacum* leaf discs treated with 0, 5, 10, 20 mM H_2_O_2_ for 1h were subjected to electrophoresis in denaturing and reducing conditions and analyzed by western blot with an antibody against the Bid protein.

**Additional file 5.** Root hair cells microscopy in white or fluorescent light. WT and overexpressing *flBid A. thaliana* roots analyzed 24 hours after 10 mM H_2_O_2_ treatment: a) and b) show an example of flBid root hair in white or fluorescent light respectively; c) and d) show a WT root hair with retraction of the cytoplasm visible in white light, the same structure is not visible following FDA treatment.

**Additional file 6.** Root hair cells analysis. a) and b) show total number of root hairs cells in control flBid and WT plants; c) and d) show changes after treatment with 10 mM H_2_O_2_. The number of root hairs alive has been evaluated with white and fluorescent light after FDA treatment while apoptotic root hair following the retraction of the cytoplasm visible in white light.

## Data Availability

All data generated or analysed during this study are included in this published article [and its supplementary information files]

## Abbreviations

PCD: Programmed Cell Death
flBid: Full-length Bid
tBid: Truncated Bid
HR: Hypersensitive Response
