# Supplementary figures and images for: Plants expressing murine pro-apoptotic protein Bid do not have enhanced PCD
Source: BMC Res Notes. 2020 Sep 21;13:450. doi: 10.1186/s13104-020-05285-x (PMC7507633; doi:10.1186/s13104-020-05285-x)

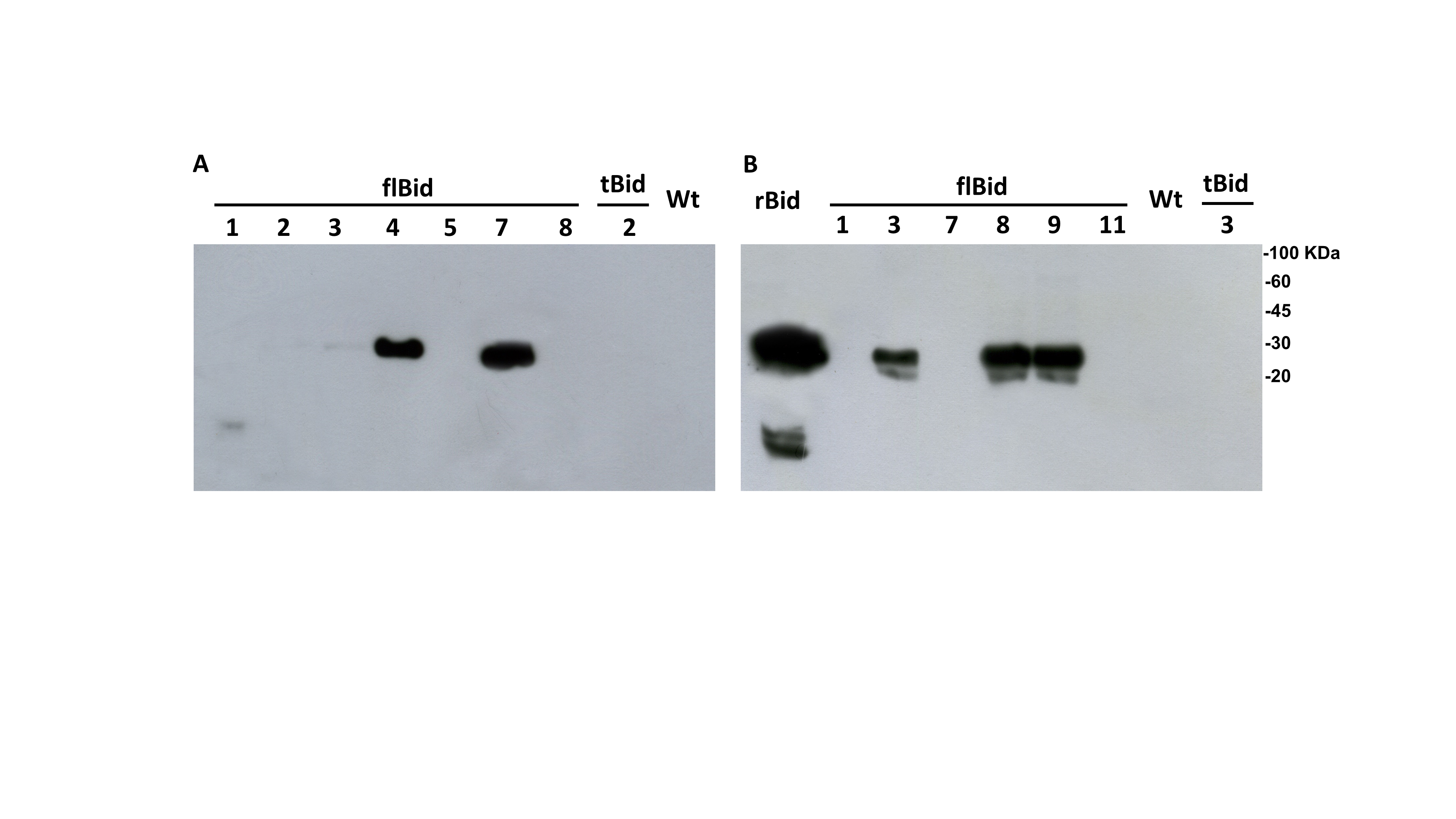

Supplement: Supplementary file 2 — Additional file 2. Western Blot analysis of flBid and tBid expression in A. thaliana (A) and N. tabacum (B) transformed plants. Numbers represent different transformed lines (1-2-3-4-5-7-8-9-11 for flBid and 2-3 for tBid); rBid: purified recombinant Bid protein; WT: wild type plants extract. [file 13104_2020_5285_MOESM2_ESM.tif]

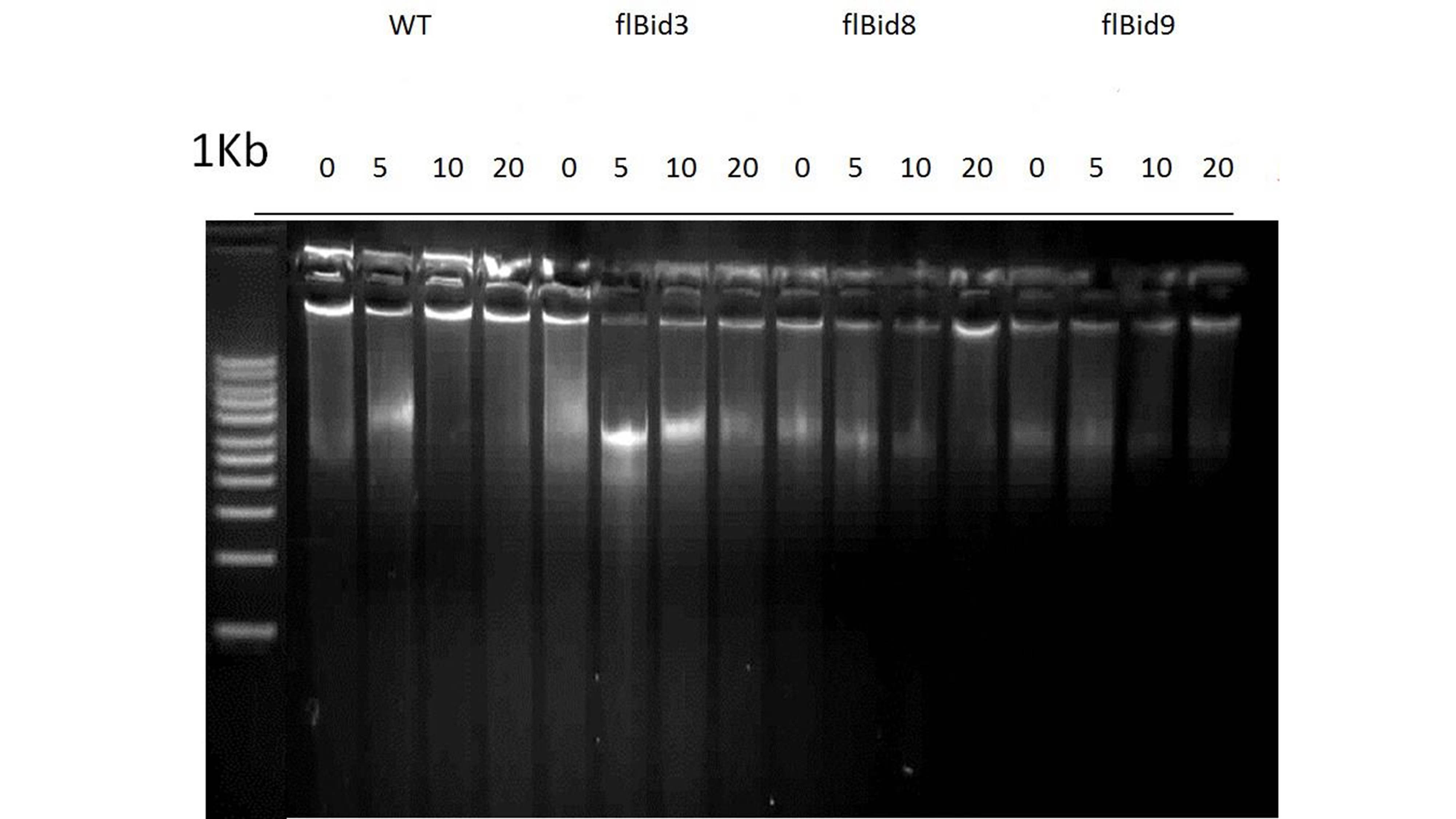

Supplement: Supplementary file 3 — Additional file 3. DNA laddering of tobacco leaf discs treated with 0, 5, 10 and 20 mM H2O2. [file 13104_2020_5285_MOESM3_ESM.tif]

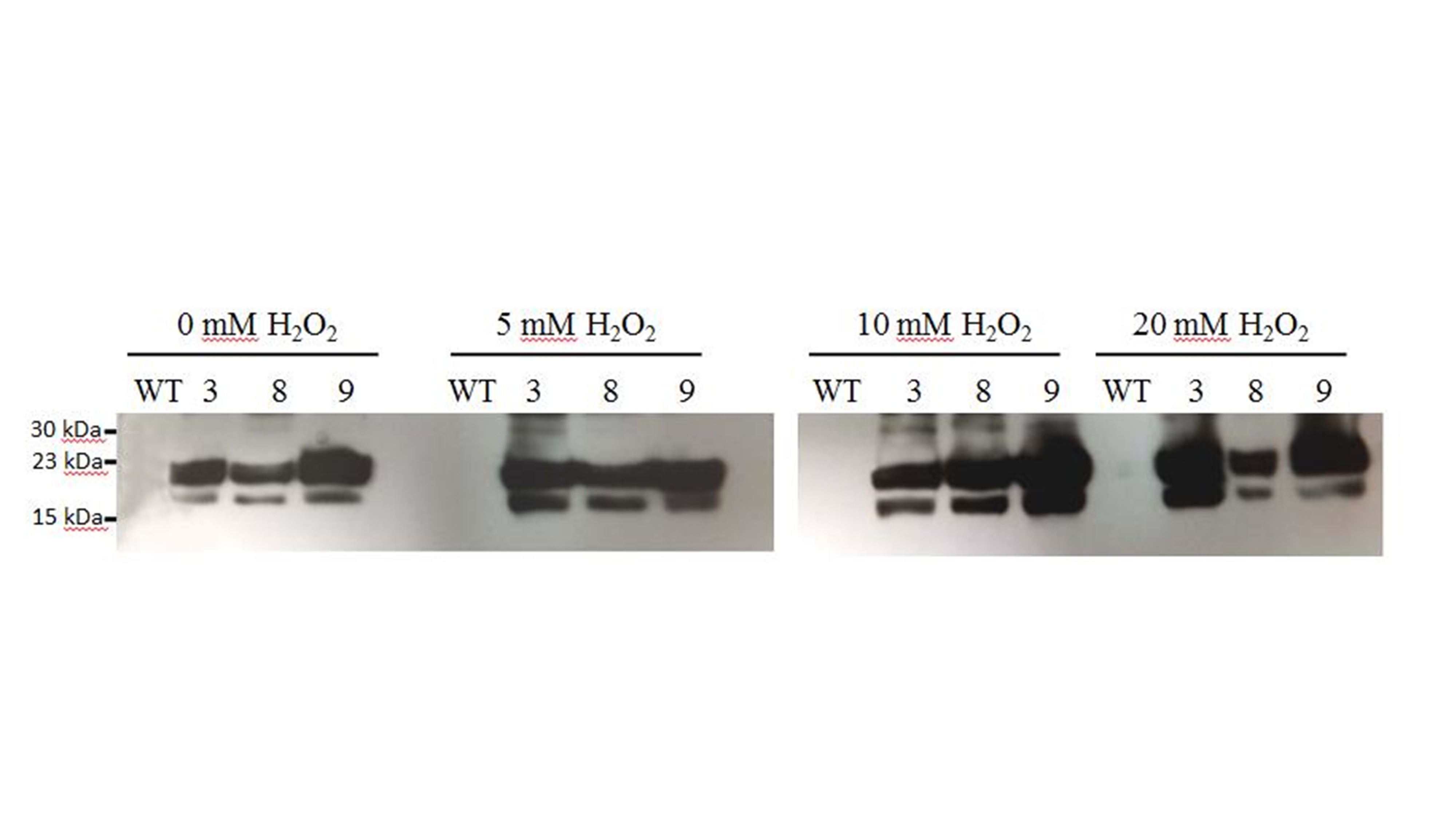

Supplement: Supplementary file 4 — Additional file 4. flBid and tBid Western blot analysis. Total proteins extracted from wild type (WT) and 35S::flBid (lines 3, 8, 9) N. tabacum leaf discs treated with 0, 5, 10, 20 mM H2O2 for 1h were subjected to electrophoresis in denaturing and reducing conditions and analyzed by western blot with an antibody against the Bid protein. [file 13104_2020_5285_MOESM4_ESM.tif]

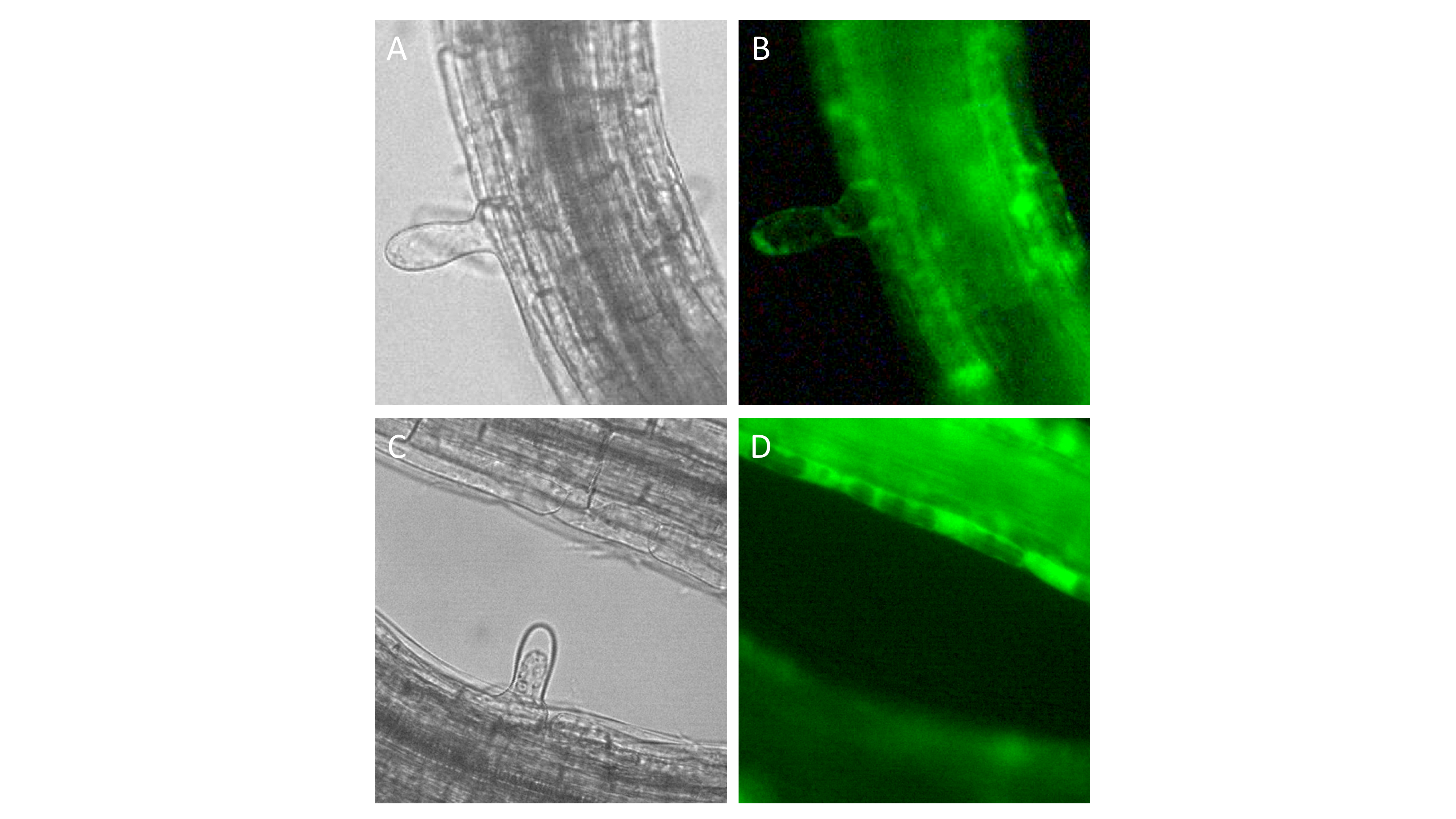

Supplement: Supplementary file 5 — Additional file 5. Root hair cells microscopy in white or fluorescent light. WT and overexpressing flBid A. thaliana roots analyzed 24 hours after 10 mM H2O2 treatment: a) and b) show an example of flBid root hair in white or fluorescent light respectively; c) and d) show a WT root hair with retraction of the cytoplasm visible in white light, the same structure is not visible following FDA treatment. [file 13104_2020_5285_MOESM5_ESM.tif]

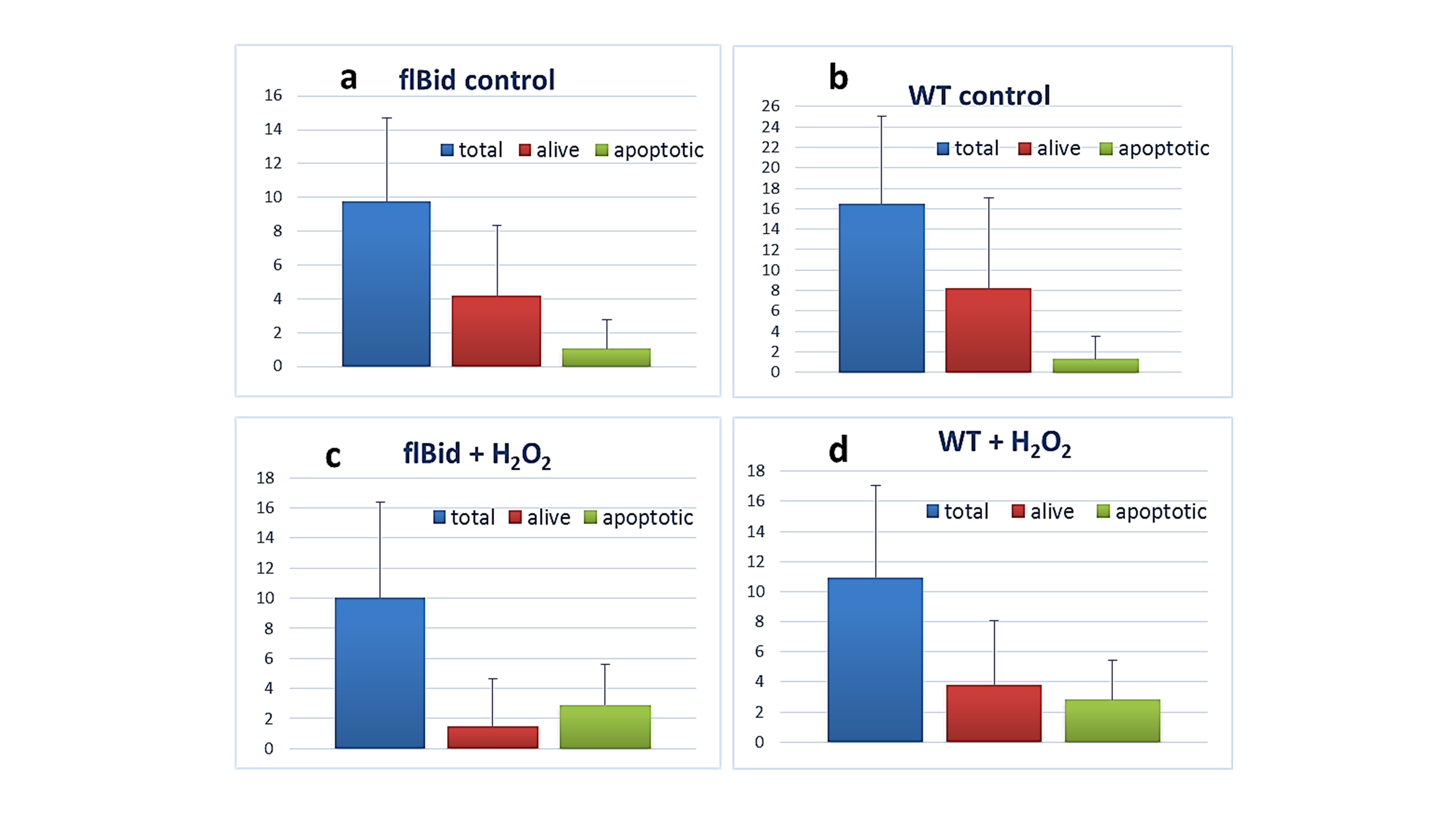

Supplement: Supplementary file 6 — Additional file 6. Root hair cells analysis. a) and b) show total number of root hairs cells in control flBid and WT plants; c) and d) show changes after treatment with 10 mM H2O2. The number of root hairs alive has been evaluated with white and fluorescent light after FDA treatment while apoptotic root hair following the retraction of the cytoplasm visible in white light. [file 13104_2020_5285_MOESM6_ESM.tif]
